# Soil Multifunctionality Differences and Driving Pathways Among Plantations in Coastal Saline–Alkali Land

**DOI:** 10.3390/plants15213491

**Published:** 2026-11-09

**Authors:** Qing Xu, Dong Han, Tao Li, Xin Liu, Jinchi Zhang

**Affiliations:** Co-Innovation Center for Sustainable Forestry in Southern China, Jiangsu Province Key Laboratory of Soil and Water Conservation and Ecological Restoration, College of Forestry and Grassland, College of Soil and Water Conservation, Nanjing Forestry University, 159 Longpan Road, Nanjing 210037, China

**Keywords:** ecological restoration, afforestation tree species, water-stable aggregates, Z-score standardization, SHAP analysis

## Abstract

Afforestation of saline–alkali tidal flats is a key ecological engineering measure to mitigate soil degradation; however, the pathways through which different plantation types drive changes in soil multifunctionality remain unclear. We selected 15-year-old stands of 10 plantation types and a bare tidal flat (CK) in the Dafeng Forest Farm of northern Jiangsu and measured 20 soil physicochemical and biological indicators in three layers (0–20, 20–40, and 40–60 cm). Z-score standardization was used to construct carbon sequestration, nutrient cycling, enzyme activity, and comprehensive multifunctionality indices, which were analyzed via two-way ANOVA, SHAP, and PLS-PM. After afforestation, topsoil electrical conductivity decreased from 4563 to 83–202 µS cm^−1^ and mean weight diameter increased from 0.17 to 0.27–0.56 mm (both *p* < 0.001). The enzyme activity Z-score differed significantly among plantation types, and the comprehensive Z-score declined significantly with soil depth. SHAP identified R_0.25_, MWD, and bulk density as the dominant drivers of the comprehensive Z-score. PLS-PM (GOF = 0.615) showed that plantation types enhanced multifunctionality mainly by reducing salinity–alkalinity (path coefficient 0.96) and improving aggregate structure, whereas soil depth exerted the largest total negative effect (−0.84). *U. thomasii* and *P. tomentosa* showed preliminary potential for improving multifunctionality in this study, which requires verification across additional sites, stand ages, and seasons.

## 1. Introduction

Coastal tidal flats constitute an important reserve of land resources in China [1]; Jiangsu Province alone possesses more than 650,000 ha of tidal flat area, accounting for approximately one quarter of the national total [2]. However, because of the high groundwater table, high groundwater mineralization, poor soil structure, and nutrient deficiency, the natural vegetation on coastal saline soils develops slowly and the ecosystem services they provide are low [3]. Under the dual pressure of climate change and population growth, how to rapidly restore the soil quality of saline–alkali lands through artificial vegetation restoration has become a major issue for coastal ecological construction and national greening.

Afforestation is widely recognized as an effective approach to ameliorating saline–alkali soils and restoring soil ecological functions. It is a systematic project consisting of field raising and land preparation, salt leaching and artificial forest construction. Compared with physical and chemical amendments, biological amelioration has the advantages of low cost, strong sustainability, and multiple ecological benefits [4]. Different types of artificial forests differ in canopy structure, root architecture, litter quality, root exudates and microbial symbioses, and their ameliorative effects on soil properties also vary. Fast-growing arbors such as *P. tomentosa* can quickly close the canopy and reduce topsoil evaporation, but they may deplete soil nutrients owing to their high nutrient demand [5]; salt-tolerant tree species such as *Q. virginiana* possess strong ion compartmentalization and osmotic adjustment abilities, but their litter decomposes relatively slowly [6,7]. Therefore, systematically comparing the soil amelioration effects of different artificial forest types under integrated land preparation and afforestation measures, and screening stand types with the functions of desalination, soil fertility improvement and soil modification, is of great practical significance for guiding the construction of coastal shelterbelts [8].

Traditional evaluations of afforestation in saline–alkali lands mostly focus on single indicators, such as the magnitude of salt reduction or organic carbon increments, which can hardly reflect the comprehensive restoration status of the soil ecosystem. As the core of terrestrial ecosystems, soil simultaneously performs multiple functions, including carbon sequestration, nutrient cycling, enzymatic catalysis, and structure maintenance. In recent years, the concept of soil multifunctionality has been proposed and widely applied, emphasizing the capacity of soil to simultaneously provide multiple ecosystem functions [9]. By standardizing and integrating multiple soil indicators into a composite index, the overall effects of different land-use types or vegetation types on soil ecosystems can be evaluated more objectively. However, studies that apply the soil multifunctionality framework to tree species screening in saline–alkali afforestation are still scarce, and quantitative analyses of the linkage among tree species, soil properties, and multifunctionality are particularly lacking.

In coastal saline–alkali lands, soil salinity–alkalinity and physical structure are considered the two core factors restricting plant growth and microbial activity. High salt stress not only directly poisons plant roots and soil microorganisms, but also further deteriorates the soil physical environment by dispersing soil colloids and destroying aggregate structure [10]. As the core units of soil structure, the formation and stabilization of water-stable aggregates depend on the input of organic binding agents, and forest litter and root exudates are important sources of these binding agents [11,12]. Theoretically, the establishment of artificial forests may enhance soil multifunctionality through two pathways: first, inhibiting evaporation via canopy shading and litter cover, and reducing topsoil salinity combined with soil preparation and leaching [13]; second, promoting aggregate formation through root growth and organic matter input [12], thereby improving soil aeration and water permeability. However, there is still a lack of quantitative verification based on causal models regarding whether desalination and structural improvement mainly act on the topsoil or extend along the soil profile, as well as the relative contributions of the two pathways and their variations with soil layers.

On this basis, taking the 10 plantation types and the saline–alkali tidal flat in the Dafeng Forest Farm of northern Jiangsu as the research objects and sampling along the 0–20, 20–40, and 40–60 cm layers, this study aimed to address the following scientific questions: (1) What are the differences among plantation types in their effects on carbon sequestration, nutrient cycling, and enzyme activity, and how do these effects vary with soil depth? (2) Which soil factors are the key drivers of changes in soil multifunctionality in saline–alkali land? (3) Through which direct or indirect pathways do plantation types and soil depth affect multifunctionality, and what are the relative contributions of each pathway? We hypothesized that afforestation enhances multifunctionality mainly through a cascade pathway of “reducing surface salinity–promoting aggregate formation–restoring microbial activity”, with the strongest effect in the surface layer and a progressive decline along the soil profile; different plantation types diverge in the magnitude of improvement because of differences in root growth and litter input. The results are intended to provide a scientific basis for the construction of shelterbelt forests and targeted soil fertility cultivation in coastal saline–alkali lands.

## 2. Results

### 2.1. Effects of Plantation Type and Soil Layer on Salinity–Alkalinity, Physical Properties, Aggregate Structure, and MBC

**Figure 1:**
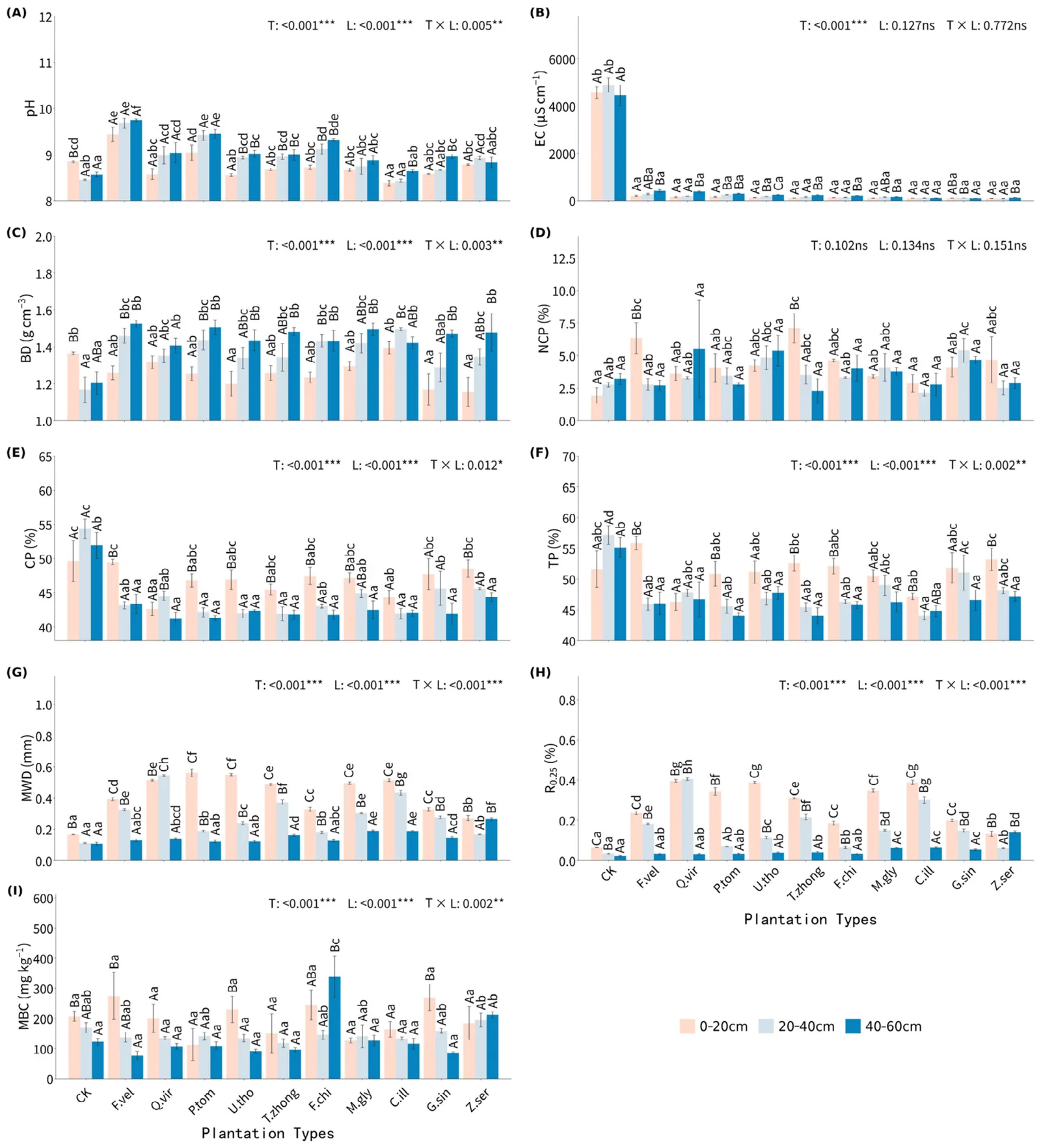
Effects of plantation type and soil layer on soil salinity–alkalinity ((**A**): pH; (**B**): EC), physical properties ((**C**): BD; (**D**): NCP; (**E**): CP; (**F**): TP), aggregate structure ((**G**): MWD; (**H**): R_0.25_), and microbial biomass carbon ((**I**): MBC). Bars are means ± SE (*n* = 3). Capital and lowercase letters indicate significant differences among soil layers and plantation types, respectively (Duncan’s multiple range test, *p* < 0.05); * *p* < 0.05, ** *p* < 0.01, *** *p* < 0.001; ns, not significant.

Two-way ANOVA showed (Figure 1) that plantation type, soil layer and their interaction had significant effects on most indicators. In terms of salinity-alkalinity, the surface soil pH of the bare tidal flat (CK) was 8.84, with an EC as high as 4563 μS·cm^−1^, and the EC in the 20–40 cm and 40–60 cm layers remained at 4453–4887 μS·cm^−1^ (Figure 1A,B). The surface soil EC of all plantations decreased significantly to 83–202 μS·cm^−1^ (plantation type effect, *p* < 0.001), among which *Z. serrata* had the lowest value (82.83 μS·cm^−1^), while the soil layer effect on EC was not significant (*p* = 0.127). The EC in the deep soil of plantations rebounded to 78–415 μS·cm^−1^; only Gleditsia sinensis and *C. illinoinensis* maintained relatively low EC values in the 40–60 cm layer. Significant differences in pH were observed among plantation types and soil layers (both *p* < 0.001, interaction *p* = 0.005): the surface soil pH of plantations ranged from 8.38 to 9.44, with *F. velutina* showing the highest pH, which further increased with soil depth. *C. illinoinensis* had the lowest surface soil pH (8.38).

For physical properties (Figure 1C–F), BD differed significantly among both plantation types and layers (both *p* < 0.001, interaction *p* = 0.003), with topsoil BD of 1.15–1.40 g cm^−3^, lowest in *U. thomasii* (1.20) and *G. sinensis* (1.17); BD generally increased with depth to 1.4–1.5 g cm^−3^ at 40–60 cm. Capillary porosity (CP) and total porosity (TP) differed significantly among plantation types and layers (both *p* < 0.05), whereas non-capillary porosity (NCP) was not significant for plantation type (*p* = 0.102), layer (*p* = 0.134), or their interaction (*p* = 0.151). For aggregate structure (Figure 1G,H), MWD of CK was only 0.17 mm and R_0.25_ only 6%; topsoil MWD of plantations increased to 0.27–0.56 mm and R_0.25_ to 13–40% (both *p* < 0.001), highest in *P. tomentosa* (0.56 mm), *U. thomasii* (0.55 mm), and *Q. virginiana* (0.51 mm); MWD and R_0.25_ decreased sharply with depth (both *p* < 0.001). MBC (Figure 1I) differed significantly among plantation types and layers (both *p* < 0.001, interaction *p* = 0.002): topsoil MBC of CK was 207 mg kg^−1^, and plantations ranged 113–274 mg kg^−1^, highest in *G. sinensis* (270), *F. velutina* (274), and *F. chinensis* (245), declining with depth.

### 2.2. Effects of Plantation Type and Soil Layer on Soil Nutrients and Carbon Fractions

**Figure 2:**
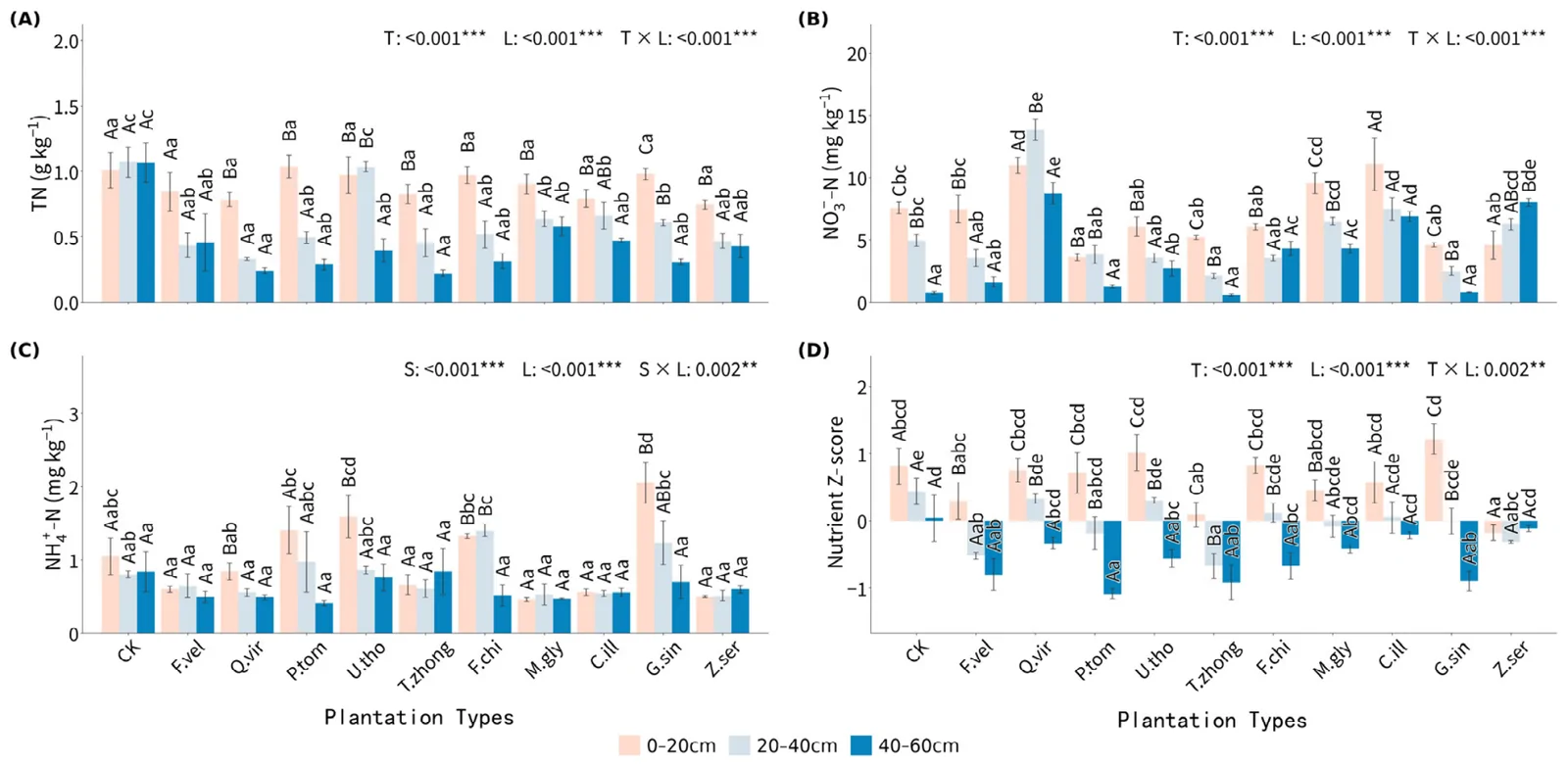
Effects of plantation type and soil layer on soil total nitrogen (**A**), nitrate N (**B**), ammonium N (**C**), and nutrient cycling Z-score (**D**). Bars are means ± SE (*n* = 3). Capital and lowercase letters indicate significant differences among soil layers and plantation types, respectively (Duncan, *p* < 0.05); ** *p* < 0.01, *** *p* < 0.001.

Figure 2 shows that total nitrogen (TN) differed extremely significantly among plantation types, soil layers, and their interaction (all *p* < 0.001), ranging from 0.75 to 1.04 g kg^−1^ and declining with depth. NO_3_^−^-N and NH_4_^+^-N differed extremely significantly among plantation types and layers (both *p* < 0.001). Topsoil NO_3_^−^-N was highest in *C. illinoinensis* (11.08 mg kg^−1^) and *Q. virginiana* (10.97 mg kg^−1^), approximately three times that of *P. tomentosa* (3.61); topsoil NH_4_^+^-N was highest in *G. sinensis* (2.05 mg kg^−1^) and lowest in *M. glyptostroboides*, *Z. serrata*, and *C. illinoinensis* (≈0.5). The nutrient group Z-score (Figure 2D) differed extremely significantly among plantation types (*p* < 0.001), highest in *G. sinensis* (1.2) and *U. thomasii* (1.0), and declined with depth.

**Figure 3:**
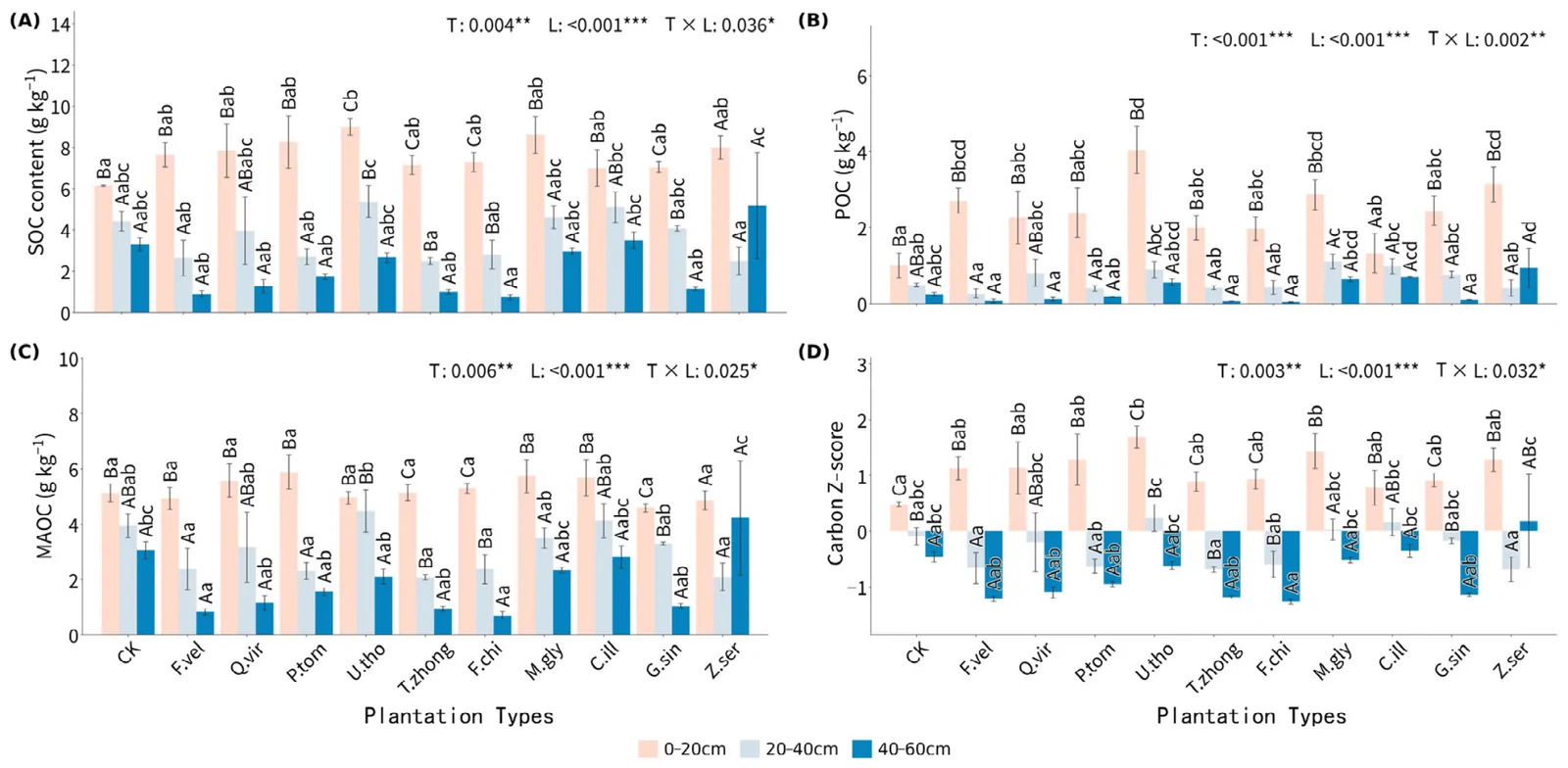
Effects of plantation type and soil layer on soil organic carbon (**A**), particulate organic carbon (**B**), mineral-associated organic carbon (**C**), and carbon sequestration Z-score (**D**). Bars are means ± SE (*n* = 3). Capital and lowercase letters indicate significant differences among soil layers and plantation types, respectively (Duncan, *p* < 0.05); * *p* < 0.05, ** *p* < 0.01, *** *p* < 0.001.

For carbon fractions (Figure 3), SOC and MAOC differed significantly among plantation types (*p* < 0.01), and POC differed extremely significantly (*p* < 0.001), all declining sharply with depth (all *p* < 0.001). Topsoil POC was lowest in CK (1.00 g kg^−1^), and *U. thomasii* (4.04) and *Z. serrata* (3.14) were significantly higher than CK by two to three times. The carbon group Z-score (Figure 3D) differed significantly among plantation types (*p* = 0.003), highest in topsoil *U. thomasii* (1.69), *M. glyptostroboides* (1.44), and *P. tomentosa* (1.28), and lowest in CK (0.48); Carbon Z generally shifted from positive to negative with depth.

### 2.3. Combined Effects of Plantation Type and Soil Layer on Soil Enzyme Activities

**Figure 4:**
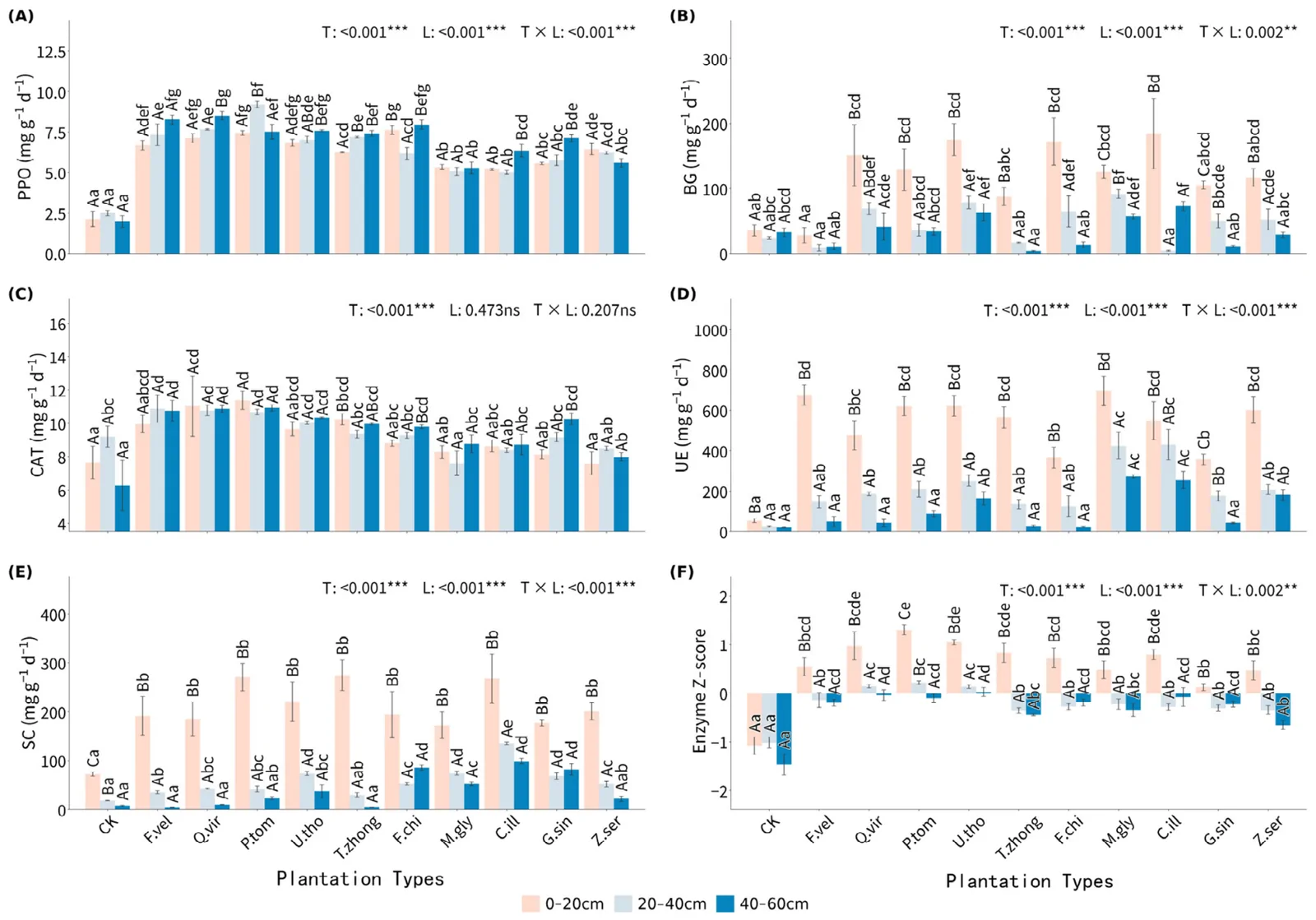
Effects of plantation type and soil layer on polyphenol oxidase (**A**), β-glucosidase (**B**), catalase (**C**), urease (**D**), sucrase (**E**), and enzyme activity Z-score (**F**). Bars are means ± SE (*n* = 3). Capital and lowercase letters indicate significant differences among soil layers and plantation types, respectively (Duncan, *p* < 0.05); ** *p* < 0.01, *** *p* < 0.001; ns, not significant.

Enzyme activities differed extremely significantly among plantation types (*p* < 0.001) and showed clear layer differentiation, but the response intensity varied among enzymes (Figure 4). PPO was lowest in CK (2.14 mg g^−1^ d^−1^), with all plantations 1.5–2.6 times higher, and highest in *F. chinensis* (7.62), *P. tomentosa* (7.45), and *Q. virginiana* (7.12). The soil layer effect (*p* = 0.473) and interaction (*p* = 0.207) on CAT were not significant, with *P. tomentosa* and *Q. virginiana* showing relatively high CAT activity. BG, UE, and SC declined significantly with depth: BG was highest in topsoil *C. illinoinensis* (184.6), significantly higher than *F. velutina* (28.2) and CK (35.5); UE was lowest in CK (21.5–53.2), with plantations 12.7–16.9 times higher, highest in topsoil *M. glyptostroboides* (696.1) and *F. velutina* (676.1); SC of all plantations (172.6–274.7) was significantly higher than CK (72.2). The enzyme group Z-score (Figure 4F) differed extremely significantly among plantation types and layers (both *p* < 0.001), with CK lowest in all three layers (−1.08 to −1.47) and topsoil highest in *P. tomentosa* (1.30), *U. thomasii* (1.05), and *Q. virginiana* (0.97), declining with depth.

### 2.4. Soil Multifunctionality and Its Key Driving Factors

**Figure 5:**
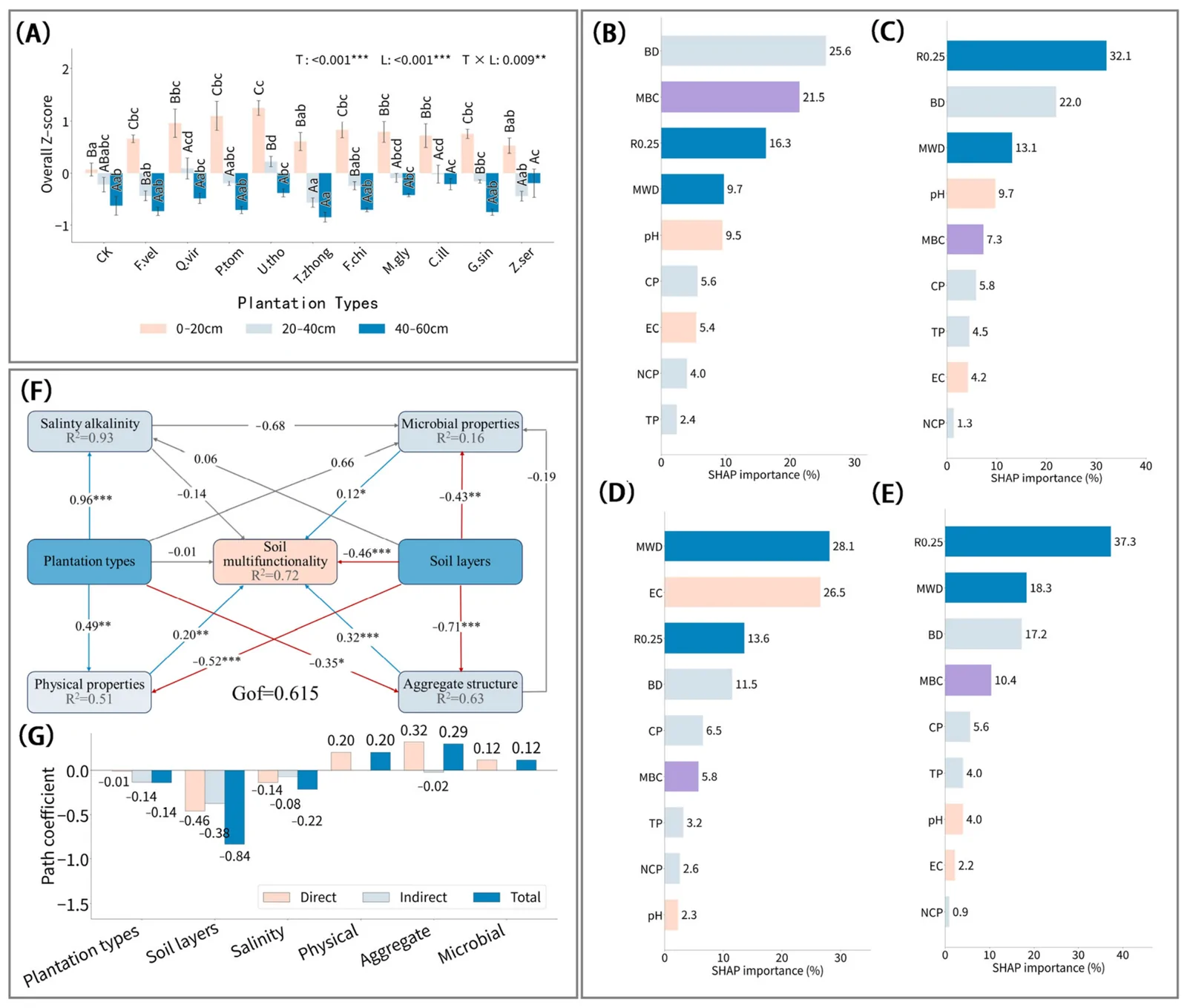
Comprehensive soil multifunctionality Z-score (**A**); relative contributions of environmental factors to nutrient cycling Z-score (**B**), carbon sequestration Z-score (**C**), enzyme activity Z-score (**D**), and comprehensive Z-score (**E**) based on SHAP; PLS-PM path model ((**F**), GOF = 0.615); direct, indirect, and total effects (**G**). In panel (**F**), blue arrows denote significant positive paths, red arrows denote significant negative paths, and gray arrows denote non-significant paths. The numbers on the arrows are standardized path coefficients. Capital and lowercase letters indicate significant differences among soil layers and plantation types, respectively (Duncan, *p* < 0.05); * *p* < 0.05, ** *p* < 0.01, *** *p* < 0.001.

Two-way ANOVA showed that the comprehensive multifunctionality Z-score (Figure 5A) differed extremely significantly among plantation types (*p* < 0.001), declined significantly with soil depth (*p* < 0.001), and showed a significant interaction (*p* = 0.009). Topsoil Overall Z was lowest in CK (0.1) and generally higher in plantations, highest in *P. tomentosa* (1.3), *U. thomasii* (1.3), and *Q. virginiana* (1.1); differences narrowed in the 20–40 cm layer, and all treatments converged at low values in the 40–60 cm layer.

SHAP analysis showed that the relative contributions of environmental factors varied with the functional target (Figure 5B–E): for the nutrient cycling Z-score (Figure 5B), BD (25.6%), MBC (21.5%), and R_0.25_ (16.3%) contributed most; for the carbon sequestration Z-score (Figure 5C), R_0.25_ (32.1%) and BD (22.0%) ranked highest; for the enzyme activity Z-score (Figure 5D), MWD (28.1%) and EC (26.5%) dominated; and for the comprehensive Z-score (Figure 5E), R_0.25_ (37.3%), MWD (18.3%), and BD (17.2%) contributed most. Overall, aggregate structure factors (R_0.25_, MWD) and BD were the main predictors of soil multifunctionality, while EC played an important role in the enzyme component.

Layer-specific SHAP analysis (Figures S3–S5) further revealed the vertical differentiation of drivers: in the 0–20 cm layer, the comprehensive Z-score was jointly dominated by MWD (22.0%), EC (19.1%), and R_0.25_ (17.7%), and MWD (27.4%) and EC (27.3%) were equally important for the enzyme Z-score, indicating that surface desalination and aggregate formation were dual drivers. In the 20–40 cm layer, the contribution of EC to the comprehensive Z-score dropped to 11.0%, replaced by pH (17.7%), R_0.25_ (15.0%), MWD (14.2%), and NCP (14.1%); pH contributed as much as 48.3% to the carbon sequestration Z-score, while EC still contributed 42.7% to the enzyme Z-score. In the 40–60 cm layer, EC contributed only 4.3% to the comprehensive Z-score, and MWD (32.1%), BD (15.2%), R_0.25_ (13.9%), and MBC (12.3%) dominated; the enzyme Z-score was driven by CP (21.6%) and MWD (18.3%). The nutrient Z-score was dominated by MBC (32.8%) and EC (26.3%) in the surface layer, shifting to MBC (24.5%) and pH (19.6%) in the deep layer.

PLS-PM (Figure 5F, GOF = 0.615) showed that plantation types extremely significantly reduced salinity–alkalinity (path coefficient 0.96, *p* < 0.001) and positively affected physical properties (0.49) and microbial properties (0.66); soil layer significantly reduced physical properties (−0.52, *p* < 0.001), aggregate structure (−0.35, *p* < 0.05), microbial properties (−0.43, *p* < 0.01), and multifunctionality (−0.46, *p* < 0.001). Physical properties (0.20, *p* < 0.01) and aggregate structure (0.32, *p* < 0.001) had significant positive direct effects on multifunctionality, while the direct effect of salinity on multifunctionality was not significant (−0.14). Effect decomposition (Figure 5G) showed that soil layer had the largest total effect on multifunctionality (−0.84), followed by physical properties (+0.32) and aggregate structure (+0.12); the direct effect of plantation type was negligible (−0.01), acting mainly indirectly through ameliorating salinity and structure.

## 3. Discussion

### 3.1. Synergistic Desalination and Structural Amelioration of Coastal Saline–Alkali Soils by Afforestation

The most notable finding of this study is that, after 15 years of afforestation, the EC of the surface soil decreased sharply from 4563 μS cm^−1^ in the tidal flat to 83–202 μS cm^−1^ in the plantations, a reduction of more than 95%, indicating that plantations in coastal saline–alkali lands have a strong desalination effect [8,12]. This result is consistent with the general understanding in coastal shelterbelt research, where raised-bed preparation combined with canopy interception and litter cover jointly suppresses capillary rise of groundwater and surface salt return, while improved soil permeability accelerates leaching of surface salts by precipitation [14,15]. Two-way ANOVA showed that the plantation type effect on EC was extremely significant (*p* < 0.001), but the soil layer effect was not significant (*p* = 0.127): the tidal flat itself showed little EC variation among layers, whereas EC rebounded in the deep layer of plantations (78–415 µS cm^−1^), with only *G. sinensis* and *C. illinoinensis* maintaining low values at 40–60 cm. This pattern indicates that the reduction in electrical conductivity was mainly confined to the surface layer, whereas salts remained at relatively high levels in the deep layers; it therefore suggests that salts may have been partly translocated downward within the profile rather than effectively removed from the soil system. Long-term monitoring of the soil salt mass balance and drainage is required to confirm whether afforestation leads to net salt export from the profile.

Accompanying desalination was a fundamental improvement in soil aggregate structure. MWD of CK was only 0.17 mm, and R_0.25_ was only 6%, indicating that the tidal flat soil lacked organic binding agents and was highly fragmented [16,17]; after afforestation, the MWD increased to 0.27–0.56 mm and R_0.25_ to 13–40%, with *P. tomentosa* and *U. thomasii* performing the best. SHAP analysis further confirmed that R_0.25_ and MWD were the most important drivers of multifunctionality (contributing 37.3% and 18.3% to the comprehensive Z-score), indicating that desalination and structural amelioration are not independent processes but are tightly coupled. Salt reduction relieves stress on microorganisms and roots [18], promoting root growth and organic carbon input; increased organic binding agents in turn promote aggregate formation, and the pore structure of aggregates accelerates salt leaching [19]. This positive feedback loop of desalination–structural improvement–further desalination may be the core mechanism by which plantations rapidly restore soil functions in saline–alkali lands.

### 3.2. Higher Sensitivity of Enzyme Activity than Carbon Sequestration and Nutrient Cycling to Afforestation

Two-way ANOVA showed that the enzyme activity Z-score differed extremely significantly among both plantation types and layers (both *p* < 0.001), and the carbon sequestration, nutrient cycling, and comprehensive multifunctionality Z-scores also reached significance among plantation types (*p* < 0.01–0.001). This indicates that although the response sensitivity of different soil functional components to afforestation varies, all generally follow the pattern of low in the tidal flat, high in the surface layer of plantations, and declining in the deep layer.

Carbon sequestration and nutrient cycling indicators (SOC, TN, etc.) are stock indicators that change slowly [20], whereas enzyme activities are flux indicators that directly reflect the instantaneous activity and metabolic intensity of the soil microbial community and are more sensitive to environmental changes, especially the relief of salt stress. The enzyme Z-score of CK was the lowest in all three layers (−1.08 to −1.47), and the PPO activity was only one-third to one-quarter of that in the plantations, indicating that high salinity severely suppressed the secretion of extracellular enzymes by microorganisms; after afforestation, the salinity decreased, and microbial activity recovered rapidly, diverging among plantation types because of differences in litter chemistry and rhizosphere effects [21].

This finding has important practical implications: enzyme activity is an earlier indicator than organic carbon and total nitrogen when evaluating afforestation effects in saline–alkali land; however, because the comprehensive Z-score was already significant at 15 years, monitoring of carbon and nitrogen pool accumulation should be synchronized after canopy closure to capture the key transition from activity recovery to pool accumulation.

### 3.3. Pathway Mechanisms by Which Tree Species Affect Soil Multifunctionality

PLS-PM revealed the cascading pathways through which plantation type and soil layer affect soil multifunctionality. Plantation type extremely significantly reduced salinity–alkalinity (path coefficient 0.96, *p* < 0.001) and positively affected physical and microbial properties [22], which is fully consistent with the extremely significant differences in MWD and R_0.25_ among plantation types [23,24]. Soil layer had significant negative paths to physical properties (−0.52), aggregate structure (−0.35), microbial properties (−0.43), and multifunctionality (−0.46), quantifying the profile decline of soil functions. Physical properties (0.20, *p* < 0.01) and aggregate structure (0.32, *p* < 0.001) had significant positive direct effects on multifunctionality, while the direct effect of salinity was not significant (−0.14), acting mainly indirectly through regulating microbes and structure [25].

Effect decomposition showed that soil layer had the largest total effect on multifunctionality (−0.84), with physical properties and aggregate structure contributing positively. It should be noted that the SHAP and PLS-PM results are not contradictory, because the two methods evaluate different aspects of the data: SHAP quantifies the predictive importance of individual environmental factors, whereas PLS-PM estimates the direct and indirect causal paths among latent variables. Accordingly, EC and aggregate indices emerged as the most important predictors in SHAP, whereas the direct path from salinity to multifunctionality in PLS-PM was not significant (−0.14), because the salinity effect was largely mediated through microbial properties and aggregate structure. Layer-specific SHAP results provide profile-scale evidence for this cascade pathway: surface EC contributed as much as 27.3% to the enzyme Z-score, echoing the strong negative regulation of salinity on microbial properties in the path model, whereas EC contributed only 4.3% to the comprehensive Z-score at 40–60 cm, indicating that salt stress mainly restricted microbial activity in the surface layer; after surface desalination, the limiting factor for deep-layer functions shifted from salinity to aggregate structure (MWD contributed 32.1% to the deep comprehensive Z) and microbial substrates (MBC contributed 24.5% to the deep nutrient Z). The anomalously high contribution of pH to the carbon Z-score in the middle layer (48.3%) may be related to the accumulation of alkaline materials at mid-depth via capillary rise after raised-bed preparation, affecting the direction of organic carbon mineralization [26,27], which requires future verification using carbonate and inorganic carbon fractions. Overall, the afforestation effects showed a clear surface-driven, profile-declining pattern: the surface layer is dominated by rapid desalination and aggregate recovery, while the deep layer depends more on long-term accumulation of root-derived organic matter and structural amelioration. From the perspective of plantation selection, *U. thomasii* and *P. tomentosa* performed best in both the comprehensive and enzyme Z-scores. The advantage of *U. thomasii* may originate from its well-developed lateral root system and high litter input, promoting aggregate formation and organic carbon accumulation; as a fast-growing species, *P. tomentosa* stimulates microbial activity and enzyme secretion through rapid root turnover and rhizodeposition [28]; however, because these root and litter traits were not directly measured in this study, these mechanisms remain hypotheses based on the literature and require direct verification in future studies.

This study has several limitations. First, the bare tidal flat control (CK) did not receive the same raised-bed preparation as the plantations, so the observed strong desalination effect may conflate the effects of raised-bed drainage, microtopographic change, and evaporation suppression, and cannot be fully attributed to the plantations themselves; a raised-bed-without-planting control is needed in future studies. Second, soil multifunctionality was constructed by global equal-weight Z-scores, which do not account for trade-offs and threshold effects among functions. Third, SHAP and PLS-PM are based on observational data and reveal statistical associations rather than strict causality; this study used space-for-time substitution at a single stand age (15 years) and single season (April 2023), and recovery dynamics across stand ages and seasons require long-term monitoring. Fourth, the nutrient cycling group comprised only TN, NO_3_^−^-N, and NH_4_^+^-N, without available phosphorus, available potassium, or cation exchange capacity; the equal-weight scheme may also mask trade-offs among functions, and sensitivity analyses of the weighting scheme are needed in future studies. Fifth, samples from different depths of the same plot are not fully independent; although soil layer was included as an explicit variable in the multivariate models and the SHAP analyses were performed separately for each layer, the model-based analyses may still be subject to pseudoreplication, and mixed-effects models with plot as a random factor are recommended when a larger number of replicate plots becomes available.

## 4. Materials and Methods

### 4.1. Study Area

The study area is located in the Dafeng Forest Farm, Yancheng City, Jiangsu Province (33°10′–33°21′ N, 120°28′–120°38′ E), on the coast of the Yellow Sea (Figure S1). It has a humid monsoon climate transitional between the northern subtropics and warm temperate zones. The mean annual temperature is 14.1 °C, the mean annual precipitation is 1042 mm, and the annual evaporation is 1335 mm, with precipitation concentrated from June to September. The soil developed from marine sedimentary parent material and is a typical coastal saline soil with a sandy texture, a groundwater table of approximately 1.0–1.5 m, and high mineralization. The plots were established within the same geomorphic unit and shared comparable environmental conditions, parent material and reclamation history [29]; hence, their initial soil properties can be considered broadly comparable, allowing the differences observed among plantation types to be attributed to the 15 years of afforestation. The plantation sites were originally mudflats formed by coastal sediment deposition and had been reclaimed into a saline–alkali ploughed field before afforestation; afforestation was carried out using the raised-bed (taitian) site preparation method in 2007–2008. The forest stand was 15 years old at sampling, and stand information is shown in Table S1.

### 4.2. Plot Design and Soil Sampling

In April 2023, a completely randomized design was adopted in the Dafeng Forest Farm, and a bare saline–alkali tidal flat (CK) and plantations of 10 major afforestation tree species were selected, with three fixed 20 m × 20 m replicate plots established for each treatment. All plots shared the same slope aspect, slope position, and elevation to eliminate site differences. Three representative sampling points were selected in an S-shape within each plot. Soil samples were collected from three layers: 0–20 cm, 20–40 cm and 40–60 cm. The samples from the three points of the same layer in the same plot were mixed into one composite sample. In the laboratory, the samples were gently broken into small clods of approximately 1 cm in diameter along natural fracture planes, and stones and plant residues were removed, after which the samples were air-dried in a shaded place for aggregate analysis. Meanwhile, approximately 1 kg of fresh soil was collected, preserved with ice packs, and transported to the laboratory, where it was stored at 4 °C and passed through a 2 mm sieve after removing stones and roots; these samples were used for the determination of active organic carbon fractions and soil enzyme activities. In addition, soil bulk density, porosity, and water content were determined using 100 cm^3^ cutting rings and aluminum boxes, and all sampling was performed with three replicates to ensure accuracy.

### 4.3. Measured Indicators

A total of 20 soil indicators were determined and classified into seven categories: (1) Saline–alkali indicators: pH and electrical conductivity (EC) were measured using a PHS-3C pH meter (Shanghai INESA Scientific Instrument Co., Ltd., Shanghai, China) and a DDS-307A conductivity meter (Shanghai INESA Scientific Instrument Co., Ltd., Shanghai, China), respectively, in a 1:5 (*w*/*v*) soil-water extract [30]. (2) Physical properties: Bulk density (BD) was determined by the oven-drying and weighing method using a 100 cm^3^ cutting ring; non-capillary porosity (NCP), capillary porosity (CP) and total porosity (TP) were calculated from the bulk density obtained by the cutting ring method and soil particle density [31]. (3) Aggregate structure: Water-stable aggregates were fractionated by wet sieving (2, 0.25, and 0.053 mm sieves, 30 oscillations min^−1^ for 30 min after 10 min pre-wetting), and MWD and R_0.25_ were calculated [29]. (4) Microbial activity: Microbial biomass carbon (MBC) was determined by the chloroform fumigation-potassium sulfate extraction method. MBC was obtained by multiplying the difference in carbon concentration between extracts of fumigated and unfumigated soil samples by the conversion coefficient kEC [32]. (5) Carbon fractions: Soil organic carbon (SOC), particulate organic carbon (POC) and mineral-associated organic carbon (MAOC) were determined by an elemental analyzer after carbonate removal via HCl fumigation [33]. POC was isolated as the >53 μm fraction after dispersion with 5 g L^−1^ sodium hexametaphosphate and sieving through a 53 μm sieve; MAOC was separated by density fractionation using 1.7 g cm^−3^ sodium iodide solution and calculated based on the mass balance of each fraction. (6) Nutrient cycling indicators: Total nitrogen (TN) was measured using a Vario EL III elemental analyzer (Elementar Analysensysteme GmbH, Langenselbold, Germany) [34]; nitrate nitrogen (NO_3_^−^-N) was determined via ultraviolet spectrophotometry (UV-2700i, Shimadzu Corporation, Kyoto, Japan) at 220 nm [35]; ammonium nitrogen (NH_4_^+^-N) was determined using the indophenol blue colorimetric method at 625 nm [35]. (7) Enzyme activity indicators [29]: Polyphenol oxidase (PPO) was assayed via the pyrogallol colorimetric method; β-glucosidase (BG) was determined via a colorimetric assay using p-nitrophenyl-β-D-glucopyranoside as the substrate; Catalase (CAT) was measured using potassium permanganate titration; Urease (UE) used urea as the substrate, and the released NH_4_^+^ from hydrolysis was quantified via the indophenol blue colorimetric method; Sucrase (SC) was determined using the 3,5-dinitrosalicylic acid (DNS) colorimetric method. The exchangeable sodium percentage (ESP) was not determined in the deep soil layers in this study and was therefore not included in the saline–alkali indicators; only its variation in the surface layer is shown in Figure S2. pH and EC were used to represent the saline–alkali status of the soil.

### 4.4. Calculation of Soil Multifunctionality

Soil multifunctionality was constructed using Z-score standardization. Global standardization (mean = 0, standard deviation = 1) was performed on all 99 samples. The mean values within the carbon group (SOC, POC, and MAOC), nutrient group (TN, NO_3_^−^-N, and NH_4_^+^-N), and enzyme group (PPO, BG, CAT, UE, and SC) were calculated to obtain Carbon Z, Nutrient Z, and Enzyme Z. The three Z values were then averaged with equal weights [36] to generate the comprehensive Z value of soil multifunctionality (Overall Z). These three functional groups were selected to represent the principal soil functions in saline–alkali afforestation systems; enzyme activities were retained as early, sensitive indicators of functional recovery, although they primarily reflect microbial metabolic activity rather than final ecosystem functions. It should be noted that the nutrient cycling Z-score was calculated only from the indicators measured in this study (TN, NO_3_^−^-N, and NH_4_^+^-N) and therefore represents nitrogen cycling rather than the full range of nutrient cycling processes (e.g., available phosphorus, available potassium, and cation exchange capacity were not included).

### 4.5. Statistical Analysis

Two-way analysis of variance (plantation types × soil layer) combined with Duncan’s multiple range test (α = 0.05) was performed, with each plot serving as the experimental unit (*n* = 3), to examine the differences in each indicator and each Z-value across 11 treatments and 3 soil layers. SHAP (SHapley Additive exPlanations) analysis based on gradient-boosted regression trees (500 trees) was used to quantify the relative contributions of nine environmental factors (pH, EC, BD, NCP, CP, TP, MWD, R_0.25_ and MBC) to the Z-values of functional groups and the integrated Z-value. The mean absolute SHAP value of each factor was expressed as the percentage of relative importance [37]. PLS-PM analysis was adopted to assess the direct and indirect effects of plantation types, soil layers, salinization (Salinity), physical properties (Physical), aggregate structure (Aggregate) and microbial biomass (Microbial) on soil multifunctionality (Overall_Z). Significance testing was conducted using 500 Bootstrap resamples, and the goodness of fit (GOF) was used to evaluate model fit [31]. All analyses were implemented in Python 3.15.2 (Python Software Foundation, Wilmington, DE, USA) and R4.6.0 (R Foundation for Statistical Computing, Vienna, Austria). To account for the hierarchical structure of the data (three soil layers nested within the same plots), soil layer was included as an explicit exogenous variable in the PLS-PM, and the SHAP analyses were performed separately for each soil layer (Figures S3–S5). Nevertheless, because samples from different depths of the same plot are not fully independent, the risk of pseudoreplication in these model-based analyses is acknowledged, and mixed-effects models with plot as a random factor are recommended for future studies with a larger number of replicate plots.

## 5. Conclusions

This study evaluated the effects of 10 plantation types on soil multifunctionality along the 0–20, 20–40, and 40–60 cm layers in the coastal saline–alkali land of northern Jiangsu. The main findings are as follows:

(1) Afforestation was associated with significant surface desalination and improved aggregate structure; however, because the control plots did not receive the same raised-bed site preparation, the desalination effect cannot be solely attributed to the plantations themselves, and comprehensive multifunctionality declined with soil depth, indicating that the ameliorative effect is concentrated in the surface layer. (2) Enzyme activity was more sensitive to afforestation than carbon sequestration and nutrient cycling; aggregate structure, bulk density, and salinity were the main drivers of comprehensive multifunctionality, and the dominant factor shifted along the profile from salinity to aggregates and microbial substrates. (3) PLS-PM showed that plantation types indirectly enhanced multifunctionality mainly by reducing salinity–alkalinity and improving aggregate structure, while soil layer significantly weakened these effects along the profile. Overall, afforestation enhanced soil multifunctionality through a cascade pathway of desalination–aggregate formation–microbial activity recovery, with a surface-driven, profile-declining pattern. Although the comprehensive Z-score differed significantly among plantation types, their effects on multifunctionality were largely indirect, and *U. thomasii* and *P. tomentosa* should be regarded only as preliminary candidate species pending verification across additional sites, stand ages, and seasons.

## Data Availability

The data supporting the findings of this study are included in the article. Further inquiries can be directed to the corresponding author.

## Supplementary Materials

The following supporting information can be downloaded at: https://www.mdpi.com/article/10.3390/plants15213491/s1, Table S1. Stand structural attributes of the ten plantations. Figure S1 Location of the study area in the coastal saline-alkali region of northern Jiangsu Province, eastern China. (A) The Yangtze River Delta region, with Jiangsu Province highlighted; (B) Jiangsu Province, with Yancheng City highlighted; (C) Yancheng City, with Dafeng District highlighted; (D) Dafeng Forest Farm, with the study plots of the 11 vegetation treatments (plot positions are schematic). Figure S2 Exchangeable sodium percentage (ESP) in the surface soil layer (0–20 cm) under different plantation types and the control (CK). Different lowercase letters indicate significant differences among plantation types at *p* < 0.05; *** *p* < 0.001. Figure S3 SHAP relative importance of nine environmental factors to the nutrient cycling (A), carbon sequestration (B), enzyme activity (C), and comprehensive (D) Z-scores in the 0–20 cm soil layer. Figure S4 SHAP relative importance of nine environmental factors to the nutrient cycling (A), carbon sequestration (B), enzyme activity (C), and comprehensive (D) Z-scores in the 20–40 cm soil layer. Figure S5 SHAP relative importance of nine environmental factors to the nutrient cycling (A), carbon sequestration (B), enzyme activity (C), and comprehensive (D) Z-scores in the 40–60 cm soil layer.
