# Supplementary material for: Soil Multifunctionality Differences and Driving Pathways Among Plantations in Coastal Saline–Alkali Land
Source: Plants (Basel). 2026 Nov 9;15(21):3491. doi: 10.3390/plants15213491 (PMC13765547; doi:10.3390/plants15213491)

Part 1 Supplementary Table

Table S1. Stand structural attributes of the ten plantations.

Species	Mean DBH (cm)	Mean height (m)	Stand density (trees hm^{-2})	Leaf area index	Root biomass (kg tree^{-1})
Fraxinus velutina	17.2 ± 1.69	10.4 ± 0.24	1125 ± 14	4.48 ± 0.03	16.1 ± 0.58
Quercus virginiana	14.61 ± 1.64	10.1 ± 0.65	1400 ± 25	4.03 ± 0.14	21.7 ± 0.93
Populus tomentosa	24.8 ± 1.82	19.8 ± 1.39	291 ± 14	1.43 ± 0.03	89.4 ± 9.30
Ulmus thomasii	16.4 ± 1.03	10.3 ± 0.24	1200 ± 25	7.08 ± 0.07	44.8 ± 1.42
Taxodium hybrid 'Zhongshanshan'	14 ± 0.61	7.4 ± 0.21	1308 ± 38	3.47 ± 0.08	6.22 ± 0.06
Fraxinus chinensis	9.56 ± 0.77	9.1 ± 0.11	3108 ± 76	1.98 ± 0.04	5.9 ± 0.04
Metasequoia glyptostroboides	13.66 ± 1.12	8.56 ± 0.23	1350 ± 25	3.17 ± 0.12	5.4 ± 0.12
Carya illinoinensis	14.29 ± 0.78	7.61 ± 0.59	442 ± 38	1.12 ± 0.06	17.0 ± 0.58
Gleditsia sinensis	11.6 ± 1.52	8.4 ± 0.71	1083 ± 38	1.78 ± 0.06	13.1 ± 0.80
Zelkova serrata	18.2 ± 1.03	11.05 ± 0.4	1658 ± 38	4.14 ± 0.41	17.7 ± 0.39

Note: Data are means ± SD (n = 3 plots per plantation)

Part 2 Supplementary Figures

Figure S1 Location of the study area in the coastal saline-alkali region of northern Jiangsu Province, eastern China. (A) The Yangtze River Delta region, with Jiangsu Province highlighted; (B) Jiangsu Province, with Yancheng City highlighted; (C) Yancheng City, with Dafeng District highlighted; (D) Dafeng Forest Farm, with the study plots of the 11 vegetation treatments (plot positions are schematic).

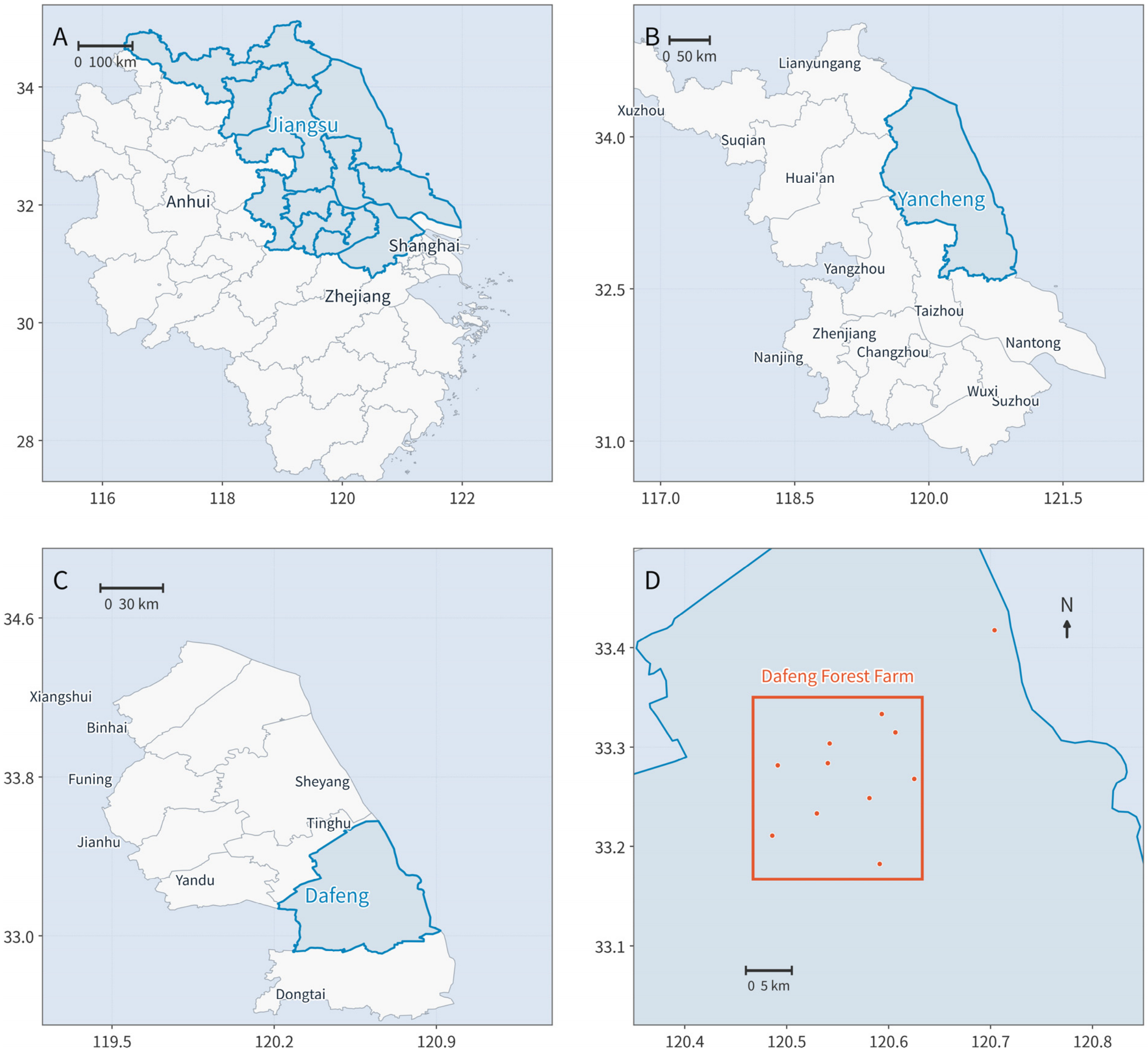

Figure S2 Exchangeable sodium percentage (ESP) in the surface soil layer (0–20 cm) under different plantation types and the control (CK). Different lowercase letters indicate significant differences among plantation types at $P < 0.05$; *** $P < 0.001$.

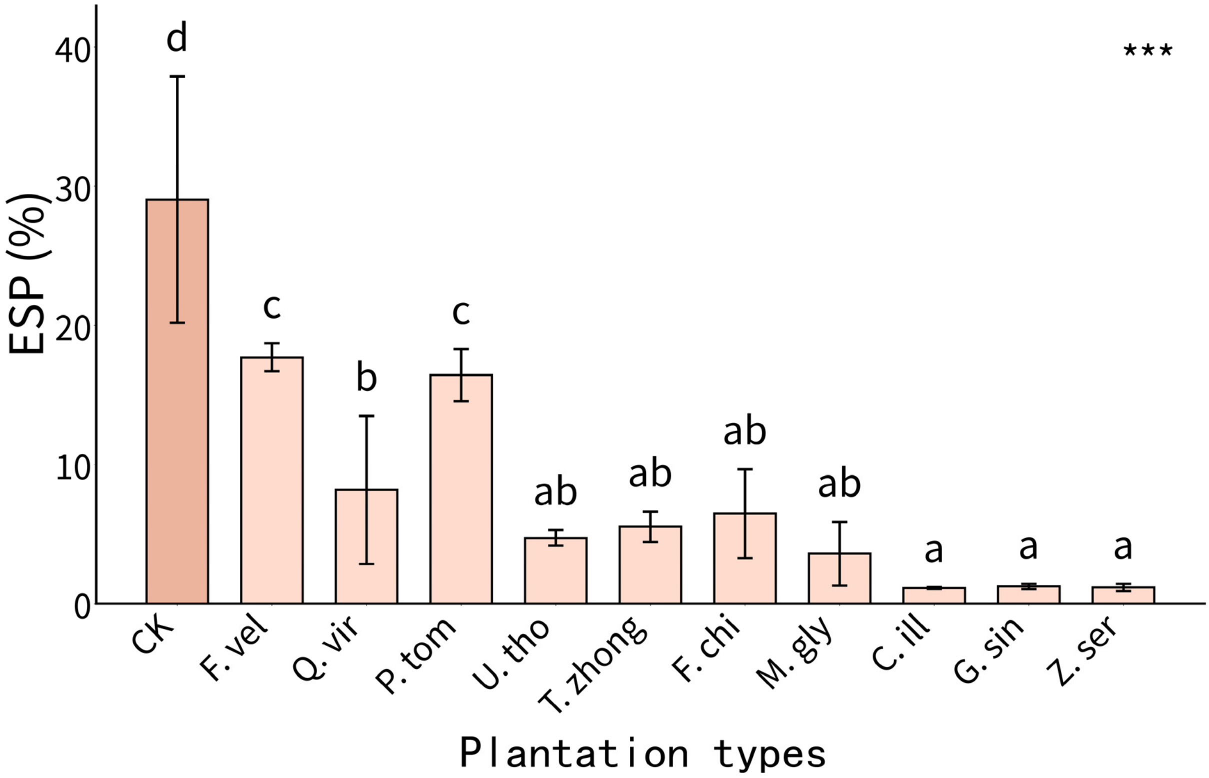

Figure S3 SHAP relative importance of nine environmental factors to the nutrient cycling (A), carbon sequestration (B), enzyme activity (C), and comprehensive (D) Z-scores in the 0–20 cm soil layer.

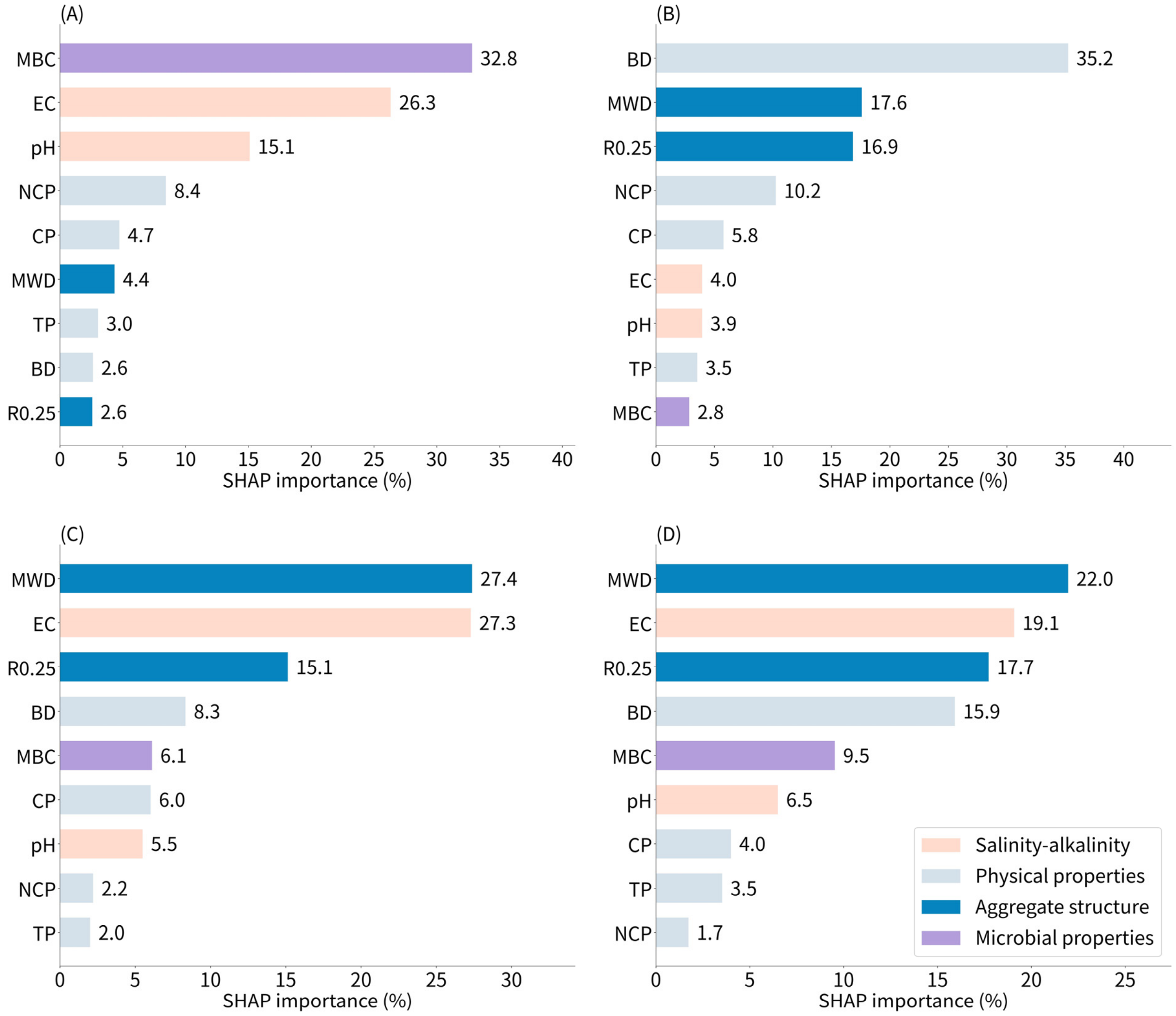

Figure S4 SHAP relative importance of nine environmental factors to the nutrient cycling (A), carbon sequestration (B), enzyme activity (C), and comprehensive (D) Z-scores in the 20–40 cm soil layer.

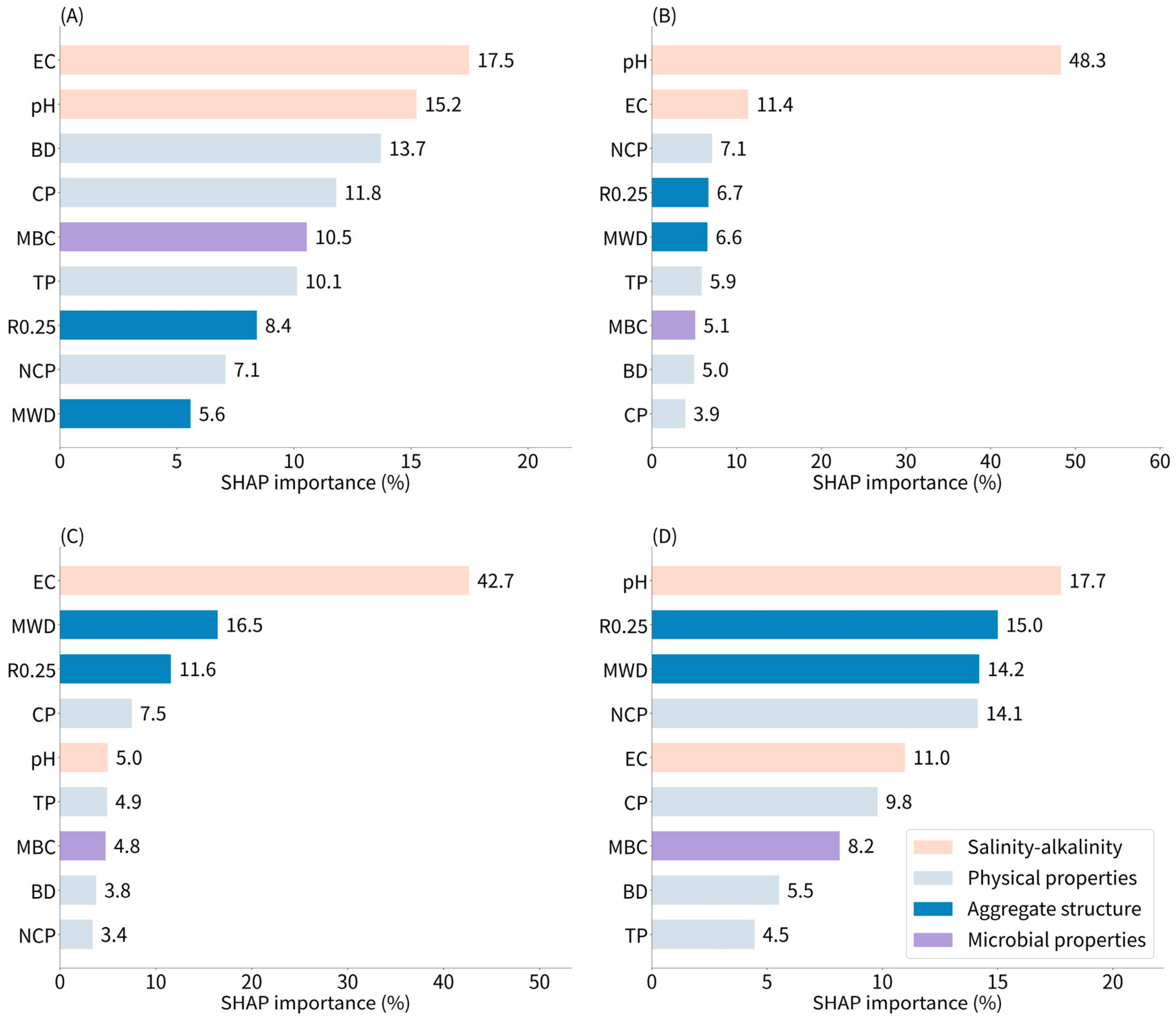

Figure S5 SHAP relative importance of nine environmental factors to the nutrient cycling (A), carbon sequestration (B), enzyme activity (C), and comprehensive (D) Z-scores in the 40–60 cm soil layer.

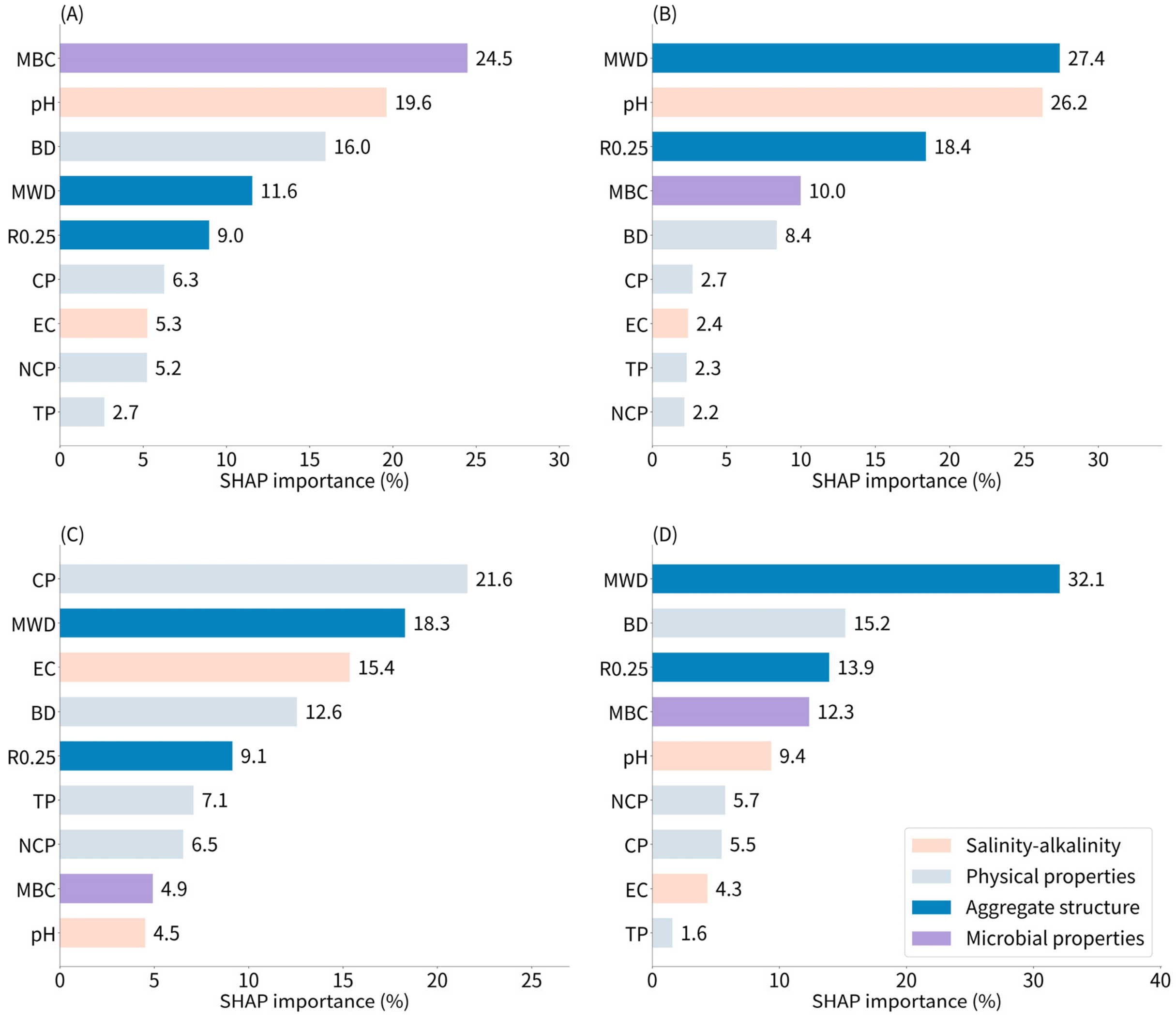

Supplement: Supplementary file 1 [file plants-15-03491-s001.zip › plants-4636683-supplementary.pdf]
